# Prevenção da Doença Cardiovascular na Adolescência: Novos Horizontes

**DOI:** 10.36660/abc.20200556

**Published:** 2021-04-08

**Affiliations:** Faculdades Pequeno Príncipe CuritibaPR Brasil Faculdades Pequeno Príncipe, Curitiba, PR – Brasil

**Keywords:** Doenças Cardiovasculares/prevenção e controle, Doenças Cardiovasculares/tendências, Adolescente, Aterosclerose, Fatores de Risco, Infância

Atualmente sabe-se que o processo de aterosclerose tem seu despertar na infância.^1^ Dessa forma, para encolher o número dos eventos cardiovasculares na população adulta, foi preciso estabelecer estratégias para evitar o desenvolvimento dos fatores de risco nas crianças.^2^ Desses, a obesidade é um entre os elementos com maior responsabilidade; sua presença e suas consequências, especialmente entre adolescentes, têm sido uma realidade cada vez mais frequente nos consultórios dos cardiologistas.^3^ Estudos vêm mostrando que o índice elevado de massa corporal significa maior probabilidade para desenvolver doenças crônicas, incluindo aterosclerose, hipertensão arterial sistêmica, diabetes melito, dislipidemia, síndrome metabólica e comorbidades como apneia obstrutiva do sono.^4^^,^^5^ Esses fatores de risco, também durante o crescimento e o desenvolvimento, tendem a se agrupar e atuam no favorecimento da doença cardiovascular.^6^

O sobrepeso e a obesidade entre crianças e adolescentes preocupam, de fato. Um olhar atento mostra que, nos últimos 20 anos, a proporção aumentou sobremaneira em vários países.^3^^,^^7^^,^^8^ A adolescência é caracterizada por mudanças significantes na composição corporal, especialmente durante a puberdade. O acompanhamento e o monitoramento são fundamentais, uma vez que peso, gordura corporal e massa magra são características preditivas na vida adulta do desenvolvimento de fatores de risco cardiovasculares.^2^^,^^4^ Essa faixa etária, dentro desse panorama, apresenta risco cinco vezes maior de adiposidade excessiva no futuro, tornando-se um marcador de risco cardiometabólico aumentado.^9^

A obesidade pode agravar outros fatores de risco. Em adolescentes, ela está associada a maiores valores de pressão arterial, principalmente, sistólica.^10^ No Brasil, os resultados do estudo ERICA mostraram que quase 1/5 da prevalência de hipertensão arterial em adolescentes no Brasil pode ser atribuída à obesidade. Segundo esse estudo, em números absolutos, cerca de 200 mil adolescentes brasileiros não teriam pressão alta se não fossem obesos.^7^ Nas crianças, os valores da pressão arterial, além de estarem associados ao sobrepeso, também se correlacionam com a distribuição de gordura corporal. Existe relação direta entre a medida da circunferência da cintura e os valores da pressão arterial.^1^

No mesmo sentido, a epidemia de obesidade infantil é também responsável pela ocorrência de doenças que impactam o metabolismo.^3^ As alterações temporais nos fatores de risco metabólico ocorrem na infância e na adolescência, anos antes do início dos eventos clínicos da doença cardiovascular.^11^ A agregação de múltiplos fatores de risco, tais como obesidade central, dislipidemia, hipertensão e resistência à insulina, entre outros, constitui a síndrome metabólica.^12^ Esta, na Pediatria, ainda é matéria controversa quanto aos seus critérios.^6^ Dessa maneira, é fundamental contextualizar que a puberdade é uma janela de tempo sensível para o desenvolvimento das origens fisiopatológicas da síndrome metabólica, visto que incorpora várias alterações hormonais e corporais. Estas incluem o acúmulo de gordura e sensibilidade reduzida à insulina que contribuem para o desfecho do estado inflamatório criado.^11^

Entendendo a obesidade, nessa população, inserida no ambiente de inflamação gerado, o processo de aterosclerose tem seu início, aceleração e progressão.^11^ A disfunção endotelial é um indicador fisiopatológico precoce e, portanto, sinaliza para o médico que assiste o adolescente a necessidade de intervir, com o objetivo de minimizar a possibilidade do aumento da morbidade e mortalidade relacionadas aos eventos do sistema cardiovascular.^11^ Mudanças patológicas e fisiológicas no endotélio vascular podem existir em crianças obesas mesmo que elas ainda não tenham desenvolvido síndrome metabólica. Devido a esse motivo, a proteção da função endotelial vascular é crucial e tornou-se alvo do tratamento dessa doença.^13^

Entre os fatores ambientais modificáveis que podem interferir no risco, o consumo da dieta obesogênica é considerado um dos principais.^3^ Contudo, outros, potencialmente plausíveis, como a curta duração do sono, vêm ganhando cada vez mais atenção nos últimos anos.^5^ A síndrome da apneia obstrutiva do sono está intimamente relacionada com ganho excessivo de peso, distúrbios metabólicos e cardiovasculares. Pacientes com apneia obstrutiva apresentam episódios hipóxicos recorrentes durante o sono, levando ao estresse oxidativo nos vasos sanguíneos e, assim, aumentam a inflamação. Muitos pesquisadores buscam investigar se o potencial negativo dos mediadores inflamatórios poderiam levar à lesão vascular sendo, então, a disfunção endotelial mediada por esse processo.^14^^,^^15^

Nesta edição dos *Arquivos Brasileiros de Cardiologia*, os autores^11^ investigam a associação entre adolescentes obesos, síndrome metabólica, disfunção endotelial e apneia obstrutiva do sono. Além disso, houve interesse em explorar a relação entre os dois últimos, uma vez que alteração endotelial é um marcador precoce de risco cardiovascular. O grupo formado pelos adolescentes obesos, quando comparado ao do constituído por adolescentes eutróficos, apresentou maior índice de circunferência abdominal, gordura corporal, pressão arterial, triglicerídios e LDLc. No mesmo sentido, encontraram menor HDLc e capacidade funcional. Foi evidenciado que 35% dos adolescentes preencheram os critérios para síndrome metabólica. Outra constatação interessante foi a associação entre disfunção endotelial e maiores valores tanto de circunferência abdominal quanto de pressão arterial sistólica. Nesse estudo, a presença da apneia obstrutiva do sono não se mostrou diferente nos dois grupos considerados. Dessa maneira, o estudo finaliza entendendo que a obesidade em adolescentes aumentou o risco para síndrome metabólica e disfunção do endotélio. Valores maiores da circunferência abdominal e pressão sistólica sustentam esse achado. Ainda, independentemente do fator obesidade, a apneia foi observada nos dois grupos.

Dessa maneira, diante do apresentado, pode-se concluir que todo o esforço é importante para a prevenção das doenças cardiovasculares do adulto. Essa começa na infância, com a identificação dos fatores de risco e abordagem precoce. A intenção é evitar a disfunção endotelial que é substrato da aterosclerose. A obesidade pode preceder distúrbios metabólicos futuros e está intimamente associada ao desenvolvimento de doenças crônicas e comorbidades.
