# Supplementary material for: Prevenção da Doença Cardiovascular na Adolescência: Novos Horizontes
Source: Arq Bras Cardiol. 2021 Apr 8;116(4):804–5. [Article in Portuguese] doi: 10.36660/abc.20200556 (PMC8121393; doi:10.36660/abc.20200556)
Supplement: Translation

# Translation: Cardiovascular Disease Prevention in Adolescence: New Possibilities

**Affiliations:** CuritibaPR Brazil Faculdades Pequeno Príncipe, Curitiba, PR – Brazil

**Keywords:** Cardiovascular Diseases/prevention and control, Cardiovascular Diseases/trends, Adolescent, Atherosclerosis, Risk Factors, Childhood

It is currently known that the atherosclerosis process starts in childhood.^1^ Thus, in order to decrease the number of cardiovascular events in the adult population, it was necessary to establish strategies aimed at preventing the development of risk factors in children.^2^ Of these, obesity is one of the most accountable elements. Obesity and its consequences, especially in adolescents, has been an increasingly frequent reality in cardiologists’ offices.^3^ Studies have shown that a high body mass index means a greater probability of developing chronic diseases, including, and among them, atherosclerosis, systemic arterial hypertension, diabetes mellitus, dyslipidemia, metabolic syndrome and comorbidities, such as obstructive sleep apnea.^4^^,^^5^ These risk factors, also during growth and development, tend to join and act in favor of cardiovascular disease.^6^

Overweight and obesity in children and adolescents are indeed a matter of concern. A close look shows that, in the last twenty years, their proportion has greatly increased in several countries.^3^^,^^7^^,^^8^ Adolescence is characterized by significant changes in body composition, especially during puberty. The follow-up and monitoring are essential since weight, body fat and lean mass are predictive characteristics of the development of cardiovascular risk factors in adult life.^2^^,^^4^ This age group, within this scenario, has a five-fold greater risk of excessive adiposity in the future, thus becoming a marker of increased cardiometabolic risk.^9^

Obesity can also aggravate other risk factors. In adolescents, it is associated with higher blood pressure, especially systolic blood pressure.^10^ In Brazil, the results of the ERICA study showed that almost 1/5 of the prevalence of hypertension in adolescents in Brazil can be attributed to obesity. According to this study, in absolute numbers, about 200,000 Brazilian adolescents would not have high blood pressure if they were not obese.^7^ In children, blood pressure levels, in addition to being associated with overweight, also correlate with body fat distribution. There is a direct association between waist circumference measures and blood pressure values.^1^

Likewise, the childhood obesity epidemic is also responsible for the occurrence of diseases that impact metabolism.^3^ Temporary alterations in metabolic risk factors occur in childhood and adolescence, years before the onset of cardiovascular disease clinical events.^11^ The aggregation of multiple risk factors, such as central obesity, dyslipidemia, hypertension and insulin resistance, among others, constitute the metabolic syndrome.^12^ This syndrome, in Pediatrics, remains a controversial matter regarding its criteria.^6^ Therefore, it is essential to contextualize that puberty is a sensitive window of time for the development of the pathophysiological origins of the metabolic syndrome, since it incorporates several hormonal and body alterations. These include the accumulation of fat and reduced insulin sensitivity, which contribute to the outcome of the established inflammatory status.^11^

Understanding obesity in this population, included in the generated inflammation environment, shows the process of atherosclerosis has its start, acceleration and progression.^11^ Endothelial dysfunction is an early pathophysiological indicator of this disease and, therefore, shows the doctor treating the adolescent that it is necessary to intervene, aiming to minimize the possibility of increased morbidity and mortality related to cardiovascular system events.^11^ Pathological and physiological alterations in the vascular endothelium may be found in obese children even if they have not yet developed metabolic syndrome. For this reason, the protection of vascular endothelial function is crucial and has become a target for the treatment of this disease.^13^

Among the modifiable environmental factors that can interfere with risk, the consumption of the obesogenic diet is considered one of the main factors.^3^ However, other potentially plausible factors, such as short sleep duration, have been gaining more and more attention in recent years.^5^ Obstructive sleep apnea syndrome is closely related to excessive weight gain, metabolic and cardiovascular disorders.^11^ Patients with obstructive apnea have recurrent hypoxic episodes during sleep, which lead to oxidative stress in the blood vessels and thus, increase inflammation. Many researchers seek to investigate whether the negative potential of inflammatory mediators could lead to vascular injury, with endothelial dysfunction then being mediated by this process.^14^^,^^15^

In this issue of the Brazilian Archives of Cardiology, the authors^11^ investigate the association between obese adolescents, metabolic syndrome, endothelial dysfunction and obstructive sleep apnea. Also, there was an interest in exploring the association between the last two, since endothelial alteration is an early marker of cardiovascular risk. The group comprised by obese adolescents, when compared to the group comprised by adolescents with normal weight, showed a higher abdominal circumference index, body fat, blood pressure, triglycerides and LDLc. Hence, they found lower HDLc levels and functional capacity. It was demonstrated that 35% of the adolescents met the criteria for metabolic syndrome. Another interesting finding was the association between endothelial dysfunction and higher values for both abdominal circumference and systolic blood pressure. In this study, the presence of obstructive sleep apnea was not different between the two assessed groups. Consequently, the study ends by assuming that obesity in adolescents increased the risk for metabolic syndrome and endothelial dysfunction. Higher values of abdominal circumference and systolic blood pressure levels support this finding. However, regardless of the obesity factor, apnea was observed in both groups.

Thus, in view of the presented facts, it can be concluded that every effort is important in the prevention of cardiovascular diseases in adults. That begins in childhood, with the identification of risk factors and early approach. The intention is to prevent endothelial dysfunction, which is an atherosclerosis substrate. Obesity can precede future metabolic disorders and is closely associated with the development of chronic diseases and comorbidities.
